# A Sultan’s Collection

**Published:** 1874-12

**Authors:** 


A Sultan's Collection.—The collection of birds and animals which the Sultan has gathered together from all parts of the world, and which includes some of the rarest specimens, has, says the *Levant Herald*, just been enriched by a choice selection of seven English dogs, presented to his Majesty by Sureya Pasha, ex-vali of Smyrna. In addition to this valuable zoological and ornithological collection, the Sultan has recently begun to form a collection of blue and white china, and emissaries are now ransacking Europe and Asia in quest of specimens fit to figure in what is designed to be the most perfect museum in the world of this branch of ceramic art.

